# Supplementary material for: Amyloid-associated increases in soluble tau relate to tau aggregation rates and cognitive decline in early Alzheimer’s disease
Source: Nat Commun. 2022 Nov 4;13:6635. doi: 10.1038/s41467-022-34129-4 (PMC9636262; doi:10.1038/s41467-022-34129-4)
Supplement: Supplementary file 1 — Supplementary Information [file 41467_2022_34129_MOESM1_ESM.pdf]

SUPPLEMENTARY INFORMATION

Amyloid-associated increases in soluble tau relate to tau aggregation rates and cognitive decline in early Alzheimer's disease

Supplementary Fig. 1. Mean spatial distribution of cross-sectional tau-PET [^{18}F]RO948 SUVR and longitudinal rate of change in individual diagnosis groups

Supplementary Fig. 2. Between group comparisons of tau-PET [^{18}F]RO948 SUVR rate of change

Supplementary Results - ADNI

Supplementary Table 1. ADNI sample characteristics

Supplementary Fig. 3. Mean spatial distribution of cross-sectional flortaucipir tau-PET SUVR and longitudinal rate of change in ADNI

Supplementary Fig. 4. Regional associations with baseline tau-PET [^{18}F]RO948 SUVR in Aβ-positive non-demented participants

Supplementary Fig. 5. CSF p-tau217 and Aβ associations using different measures of Aβ with regional tau-PET [^{18}F]RO948 rate of change in Aβ-positive non-demented participants

Supplementary Fig. 6. Regional Aβ-PET and CSF p-tau217 associations with regional tau-PET [^{18}F]RO948 rate of change in Aβ-positive CU and MCI separately

Supplementary Fig. 7. CSF p-tau181 and Aβ associations with regional tau aggregates accumulation in Aβ-positive non-demented participants in ADNI

Supplementary Fig. 8. Mediating effect of CSF p-tau217 on Aβ-PET and regional tau-PET [^{18}F]RO948 rate of change in Aβ-positive non-demented participants

Supplementary Fig. 9. Individualized connectivity-based associations of tau-PET rate of and CSF p-tau217 in CU and MCI Aβ-positive participants separately

Supplementary Fig. 10. Individualized connectivity-based associations of tau-PET rate of change over time and CSF p-tau181 in Aβ-positive non-demented participants in ADNI

Supplementary Fig. 11. Cognitive decline analyses in Aβ-positive non-demented participants using tau-PET change in the temporal meta-ROI as mediator

Supplementary Fig. 12. Cognitive decline analyses in Aβ-positive CU and MCI separately

Supplementary Fig. 13. Associations between CSF p-tau217 and Aβ-PET across the AD continuum

Supplementary Table 2. Bivariate correlations in Aβ-positive non-demented participants

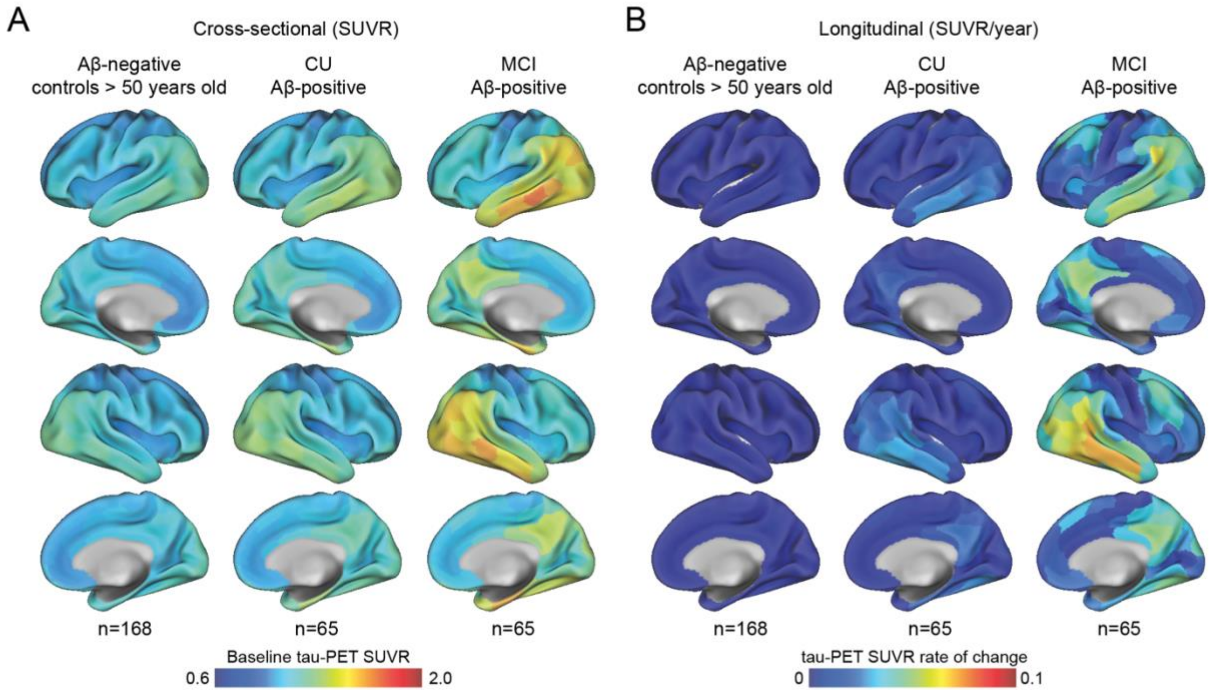

Supplementary Fig. 1. Mean spatial distribution of cross-sectional tau-PET [^{18}F]RO948 SUVR and longitudinal rate of change in individual diagnosis groups

(**A**) Surface renderings of average baseline tau-PET SUVR in Aβ-negative controls above 50 years old, Aβ-positive CU and Aβ-positive MCI in the 200 parcels from the Schaefer 200-ROI atlas (**B**) Surface renderings of yearly tau-PET SUVR rate of change derived as the slope from linear mixed-effect models in the same participants group as in (A). CU: Cognitively unimpaired; MCI: Mild cognitive impairment

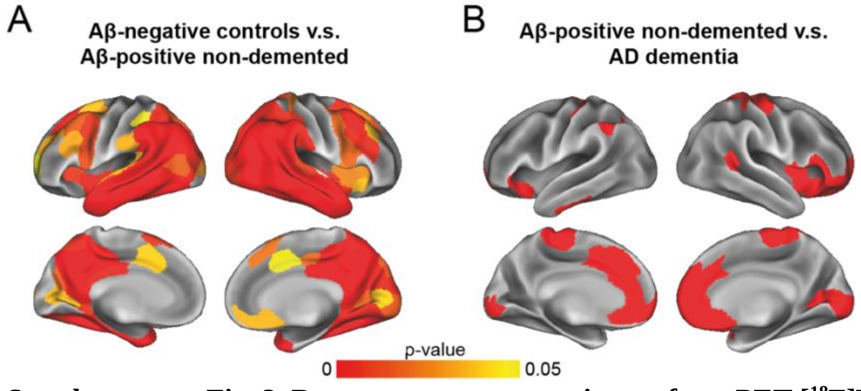

Supplementary Fig. 2. Between group comparisons of tau-PET [^{18}F]RO948 SUVR rate of change

Regions where tau-PET rate of change is significantly different between **(A)** Aβ-negative controls vs. Aβ-positive non demented participants, and **(B)** Aβ-positive non demented participants vs. Aβ-positive AD dementia patients. Two-sided t-tests were done to compare groups and regions with significant p-value from post-hoc Tukey HSD are reported on the brains.

Supplementary Results - ADNI

In the ADNI cohort, given the smaller sample size, only the results that could be validated with sufficient power (80% and alpha=0.05) were conducted. As such, the regional analyses relating soluble p-tau to local accumulation of tau aggregates were conducted, as well as the connectivity-based analyses on tau aggregates accumulation. Only 28 ADNI participants had longitudinal cognitive assessments, which was insufficient to test the mediations as done in BioFINDER-2. Based on effect sizes determined in BioFINDER-2, sample sizes between 75 and 82 participants would have been needed in order to assess effects of soluble p-tau on cognitive decline. Similarly, given the very few AD dementia patients with longitudinal tau-PET in ADNI (n=3), analyses pertaining to the dementia stage could not be conducted in this cohort.

	**Aβ-negative controls (n=52)**	**Aβ-positive non-demented (n=67)**
**Age (years)**	71.7 (6.9)	74.5 (6.7)
**Sex F (%F)**	21 (40%)	28 (42%)
**Education (years)**	16.7 (2.4)	16.6 (2.3)
***APOE*ε4 carriers** (%)	13 (25%)	42 (63%)
**CSF p-tau181** (pg/ml)	20.33 (6.98)	33.54 (16.85)
**MMSE**	29.3 (1.0)	28.4 (1.9)
**Aβ Centiloid**	-3.8 (13.8)	77.4 (33.6)
**tau-PET follow-up time** (years)	2.3 (1.1)	1.7 (0.6)

Supplementary Table 1. ADNI sample characteristics

Data are presented as mean ± standard deviation unless specified otherwise.

Abbreviations: Aβ= beta-amyloid; *APOE*ε4= apolipoprotein E genotype (carrying at least one ε4 allele); CSF p-tau181= cerebrospinal fluid phosphorylated tau 181; MMSE= Mini-Mental State Evaluation; PET= positron emission tomography.

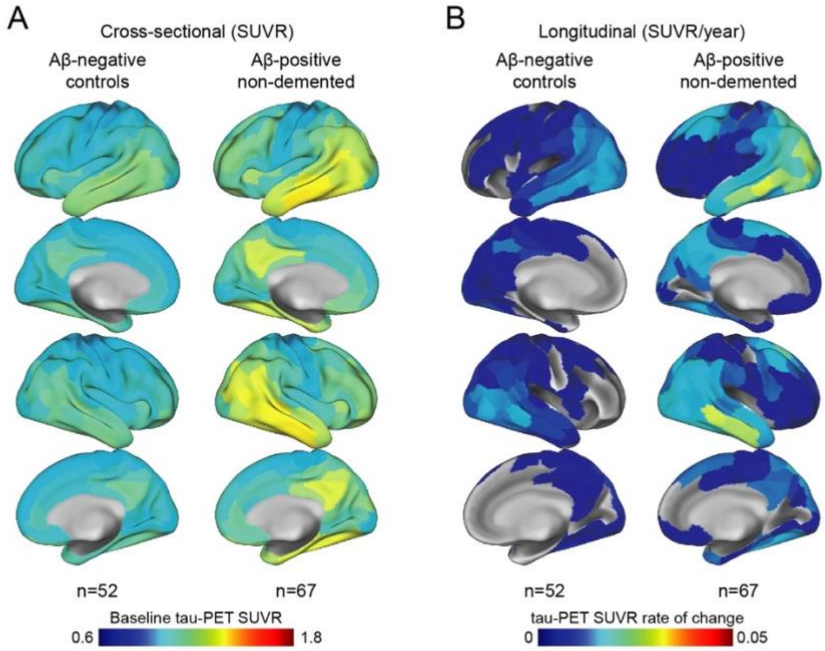

Supplementary Fig. 3. Mean spatial distribution of cross-sectional flortaucipir tau-PET SUVR and longitudinal rate of change in ADNI

(A) Surface renderings of average baseline tau-PET SUVR in Aβ-negative controls and Aβ-positive non-demented participants in the 200 parcels from the Schaefer 200-ROI atlas (B) Surface renderings of yearly tau-PET SUVR rate of change derived as the slope from linear mixed-effect models in the same participants group as in (A)

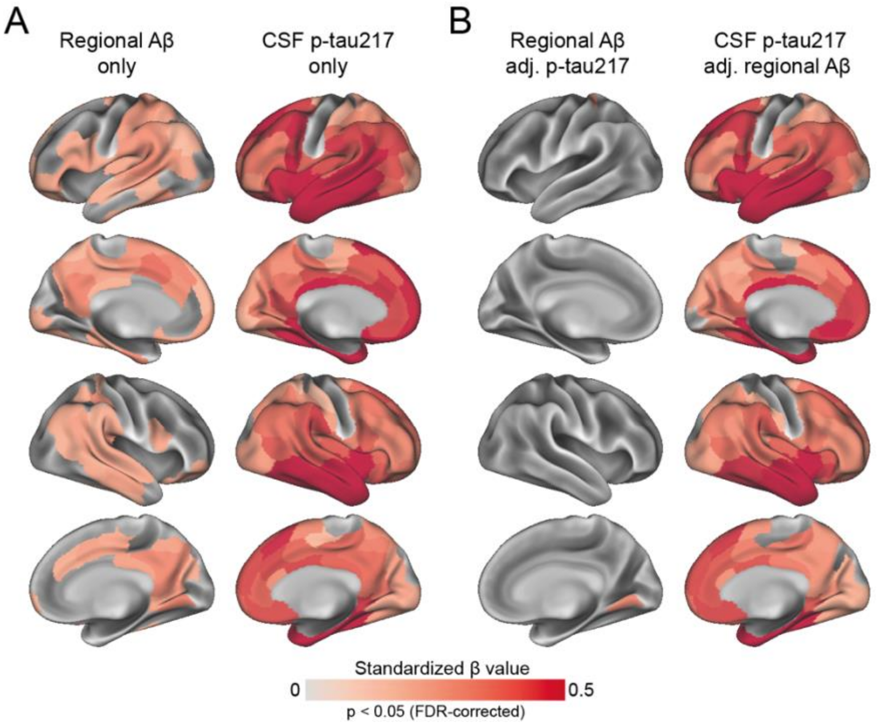

Supplementary Fig. 4. Regional associations with baseline tau-PET [^{18}F]RO948 SUVR in Aβ-positive non-demented participants

(**A**) Standardized beta coefficient of local Aβ-PET in regions where regional Aβ-PET flutemetamol SUVR (left column) relates to baseline tau-PET, adjusting for age and sex. Right column was derived from a similar model, but using CSF p-tau217 as predictor instead of Aβ-PET (**B**) Standardized beta coefficient where local Aβ-PET (left column) and CSF p-tau217 (right column) is associated to baseline tau-PET when including both biomarkers in the same model, adjusting for age and sex (tau PET ~ regional Aβ-PET + CSF p-tau217 + age + sex). All regions shown on the brain are significant at $p<0.05$ after FDR-correction from two-sided statistical tests.

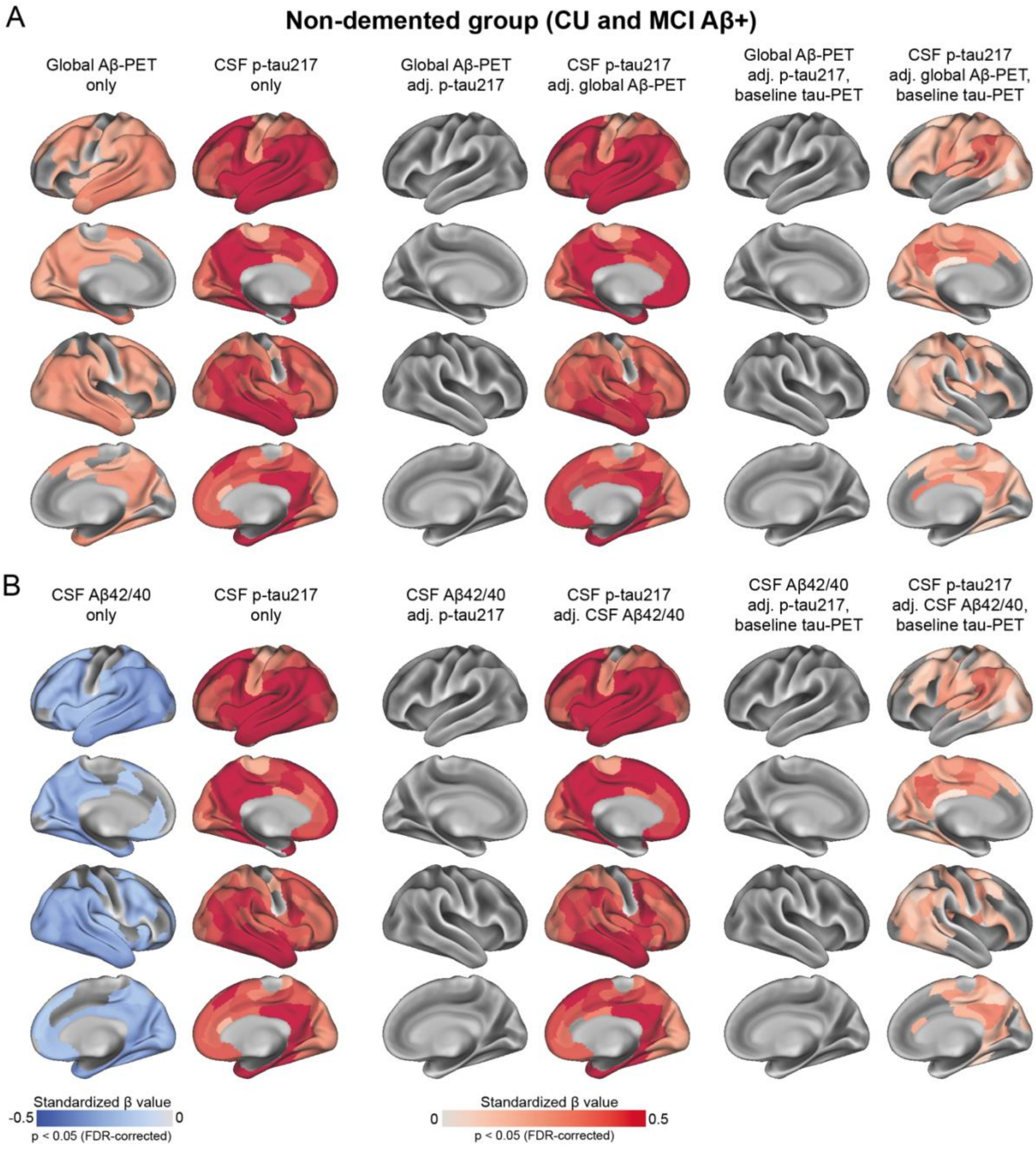

Supplementary Fig. 5. CSF p-tau217 and Aβ associations using different measures of Aβ with regional tau-PET [^{18}F]RO948 rate of change in Aβ-positive non-demented participants
Similar analyses as done on Fig. 2, using different amyloid measure instead of instead of regional Aβ-PET. The amyloid measure is global Aβ-PET SUVR in **A** and CSF Aβ42/40 ratio in **B**. In both panels, the left two columns show standardized beta coefficient in regions where Aβ and CSF p-tau respectively relates to regional tau-PET rate of change, adjusting for age and sex. Two middle columns show standardized beta coefficient where Aβ and CSF p-tau217 are associated to regional tau-PET rate of change when including both biomarkers in the same model, adjusting for age and sex (tau PET rate of change ~ Aβ + CSF p-tau217 + age + sex). Right two columns show the same depiction as in middle columns when additionally controlling for regional baseline tau-PET SUVR. All regions shown on the brain are significant at $p<0.05$ after FDR-correction from two-sided statistical tests.

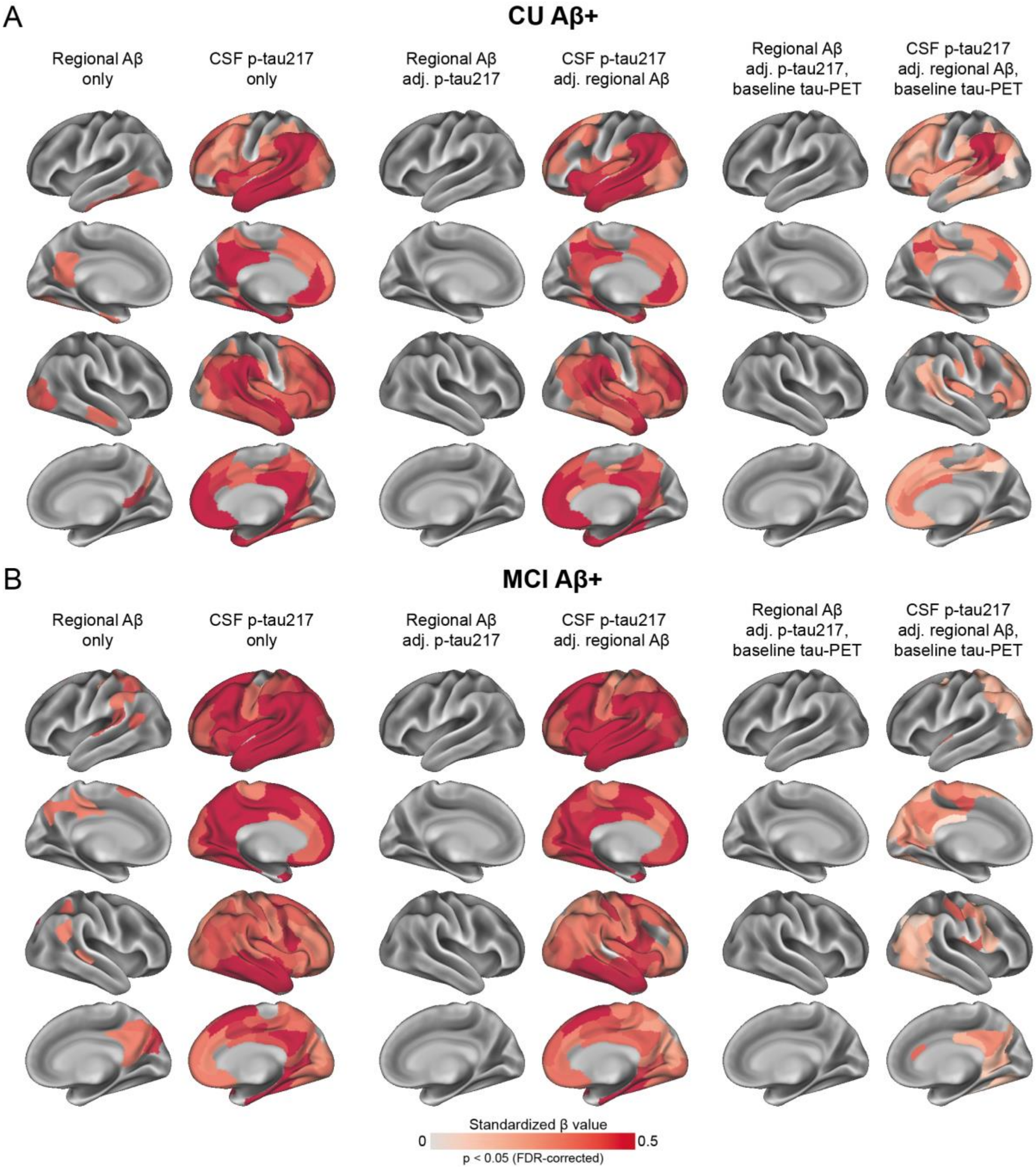

Supplementary Fig. 6. Regional Aβ-PET and CSF p-tau217 associations with regional tau-PET [18F]RO948 rate of change in Aβ-positive CU and MCI separately

Same analyses as reported in Fig. 2 in the full sample of non-demented Aβ-positive participants, when splitting the group into CU only **(A)** and MCI participants only **(B)**. In both panels, the left two columns show standardized beta coefficient in regions where regional Aβ-PET and CSF p-tau respectively relates to regional tau-PET rate of change, adjusting for age and sex. Two middle columns show standardized beta coefficient where regional Aβ-PET and CSF p-tau217 are associated to regional tau-PET rate of change when including both biomarkers in the same model, adjusting for age and sex (tau PET rate of change ~ regional Aβ-PET + CSF p-tau217 + age + sex). Right two columns show the same depiction as in middle columns when additionally controlling for regional baseline tau-PET SUVR. All regions shown on the brain are significant at $p<0.05$ after FDR-correction from two-sided statistical tests.

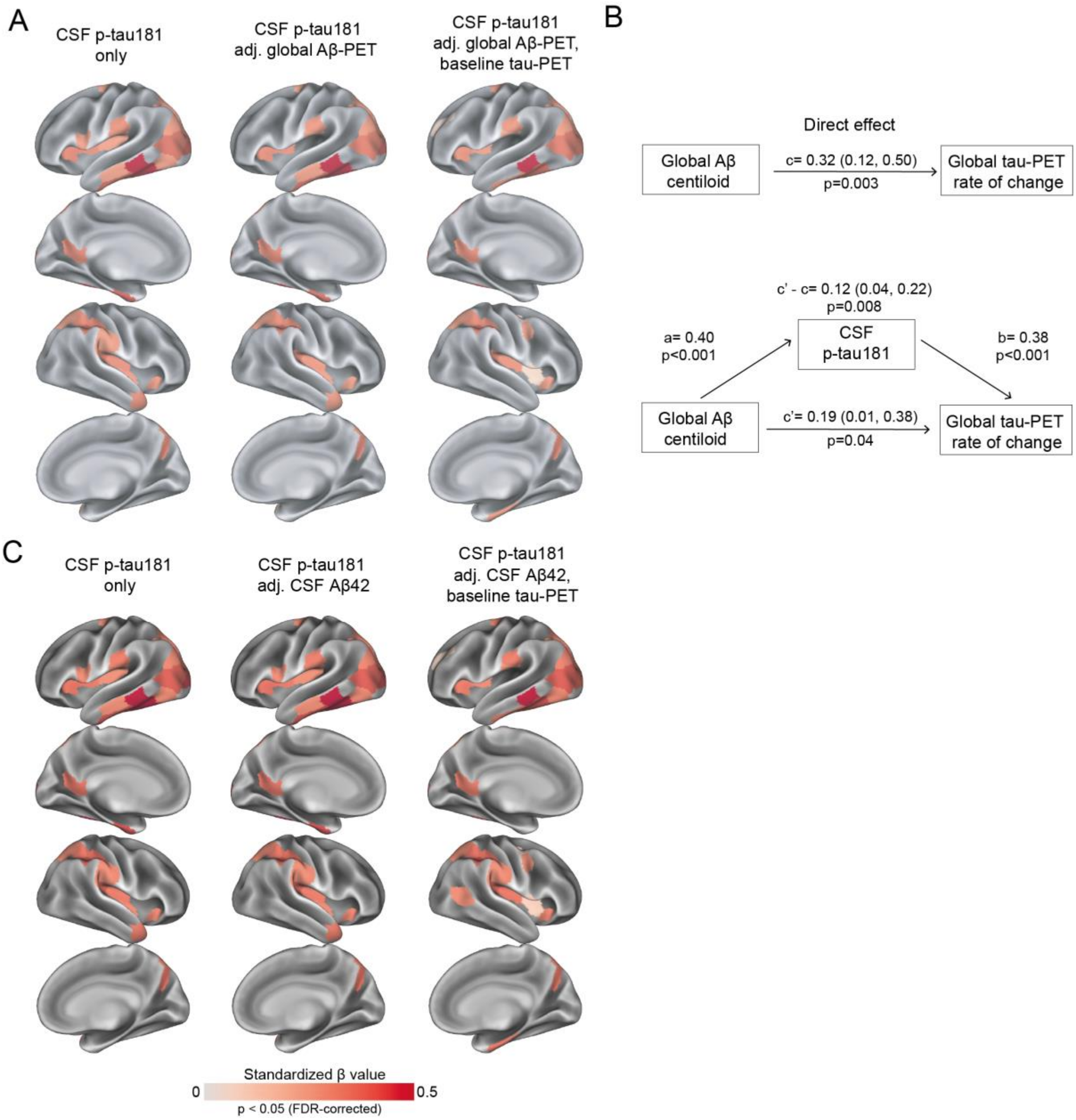

Supplementary Fig. 7. CSF p-tau181 and Aβ associations with regional tau aggregates accumulation in Aβ-positive non-demented participants in ADNI

(A) Standardized beta coefficient of CSF p-tau181 in regions where CSF p-tau181 relates to regional tau-PET rate of change, from linear regressions adjusting for age and sex (left). Standardized beta coefficient of CSF p-tau181 in regions where CSF p-tau181 relates to regional tau-PET rate of change, when adjusting for global Aβ centiloid value, age and sex (middle), and when also adjusting for regional baseline tau-PET SUVR (right). **(B)** Mediating effect of CSF p-tau181 on global Aβ centiloid value accumulation of tau aggregates across the brain (average rate of change across 200 regions). **(C)** Same analyses as shown in A, but when using CSF Aβ42 as the amyloid measure. All regions shown on the brain are significant at $p<0.05$ after FDR-correction from two-sided statistical tests.

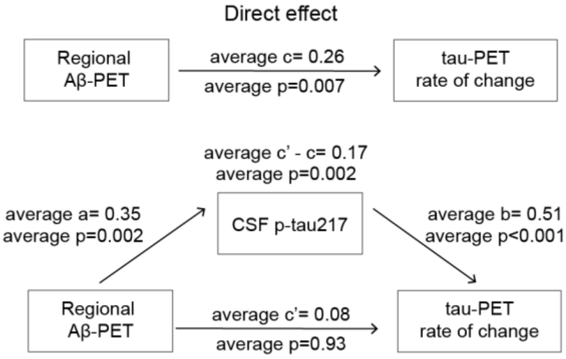

Supplementary Fig. 8. Mediating effect of CSF p-tau217 on Aβ-PET and regional tau-PET [^{18}F]RO948 rate of change in Aβ-positive non-demented participants
Summary statistics of the mediation from Fig. 2d. The coefficients and p-values reported are from the average across regions where CSF p-tau217 has a significant mediating effect, from two-sided statistical tests without adjustment for multiple comparisons.

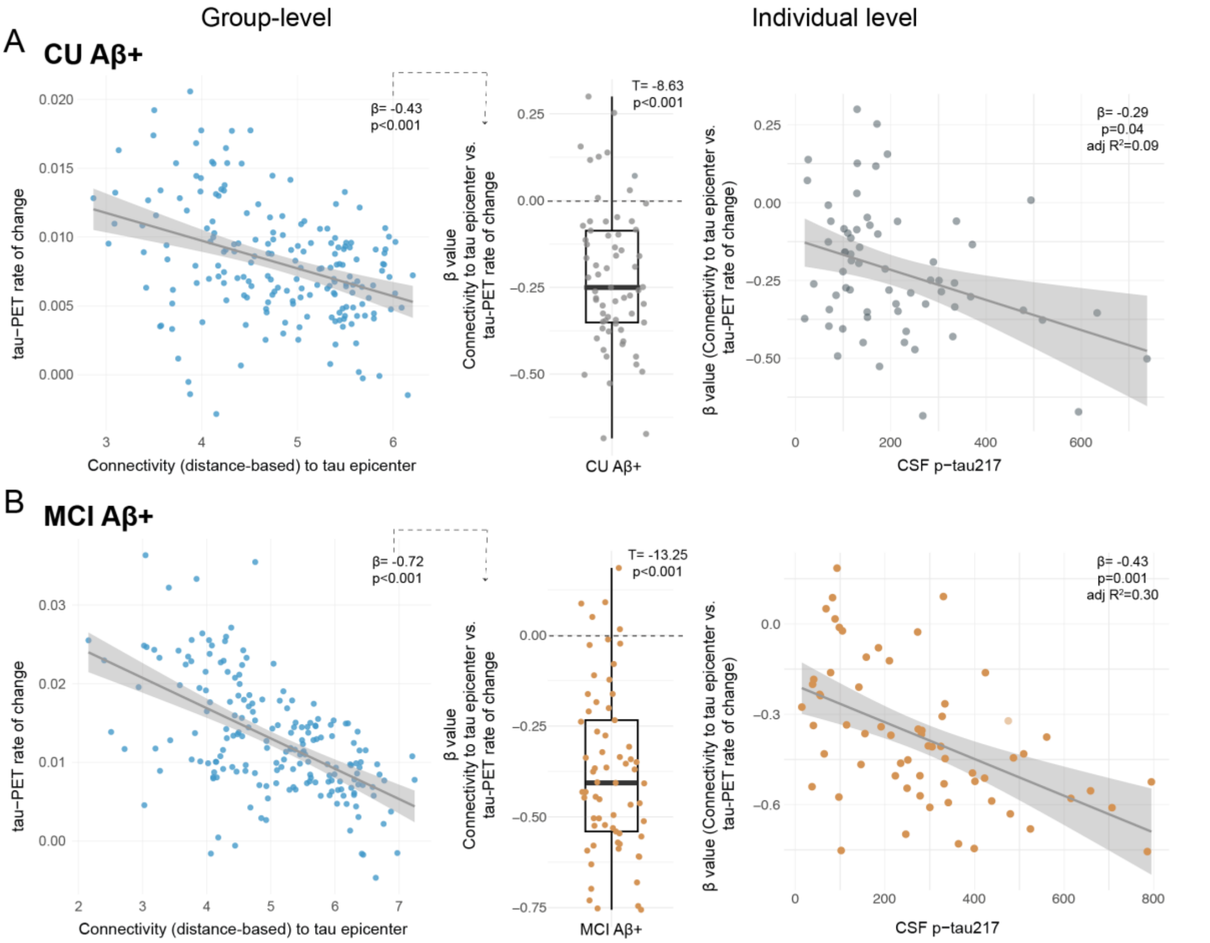

Supplementary Fig. 9. Individualized connectivity-based associations of tau-PET rate of and CSF p-tau217 in CU and MCI Aβ-positive participants separately

Same analyses as on Fig. 3A-C in the full sample of non-demented Aβ-positive participants, done here in CU Aβ-positive only **(A)** and MCI Aβ-positive **(B)**. All analyses were significant in CU (n=65) and MCI (n=65) separately. In both panels, left scatter plot depicts group-level analysis showing how connectivity to the tau epicenters relates to tau-PET rate of change across the whole brain. Each dot represents a brain region. Regions more strongly functionally connected to the epicenters have greater rate of tau-PET accumulation. Repeating the same approach depicted at the individual level, we could generate for each participant a β-value from the correlation between tau-PET rate of change and connectivity-based distance to epicenters across all brain regions, as shown on the box plot in the middle. Right scatter plots show the association between CSF p-tau217 and the β-values of epicenter connectivity to tau-PET rate of change. Each dot represents an individual. The expected negative association suggests that higher CSF p-tau217 is associated with the overall pattern of tau-PET change in more functionally connected regions to epicenters. All linear regressions performed were two-sided, without adjustment for multiple comparisons and error bands correspond to the 95% confidence interval. In all box plots, the box limits represent the interquartile range and the line depicts the median value.

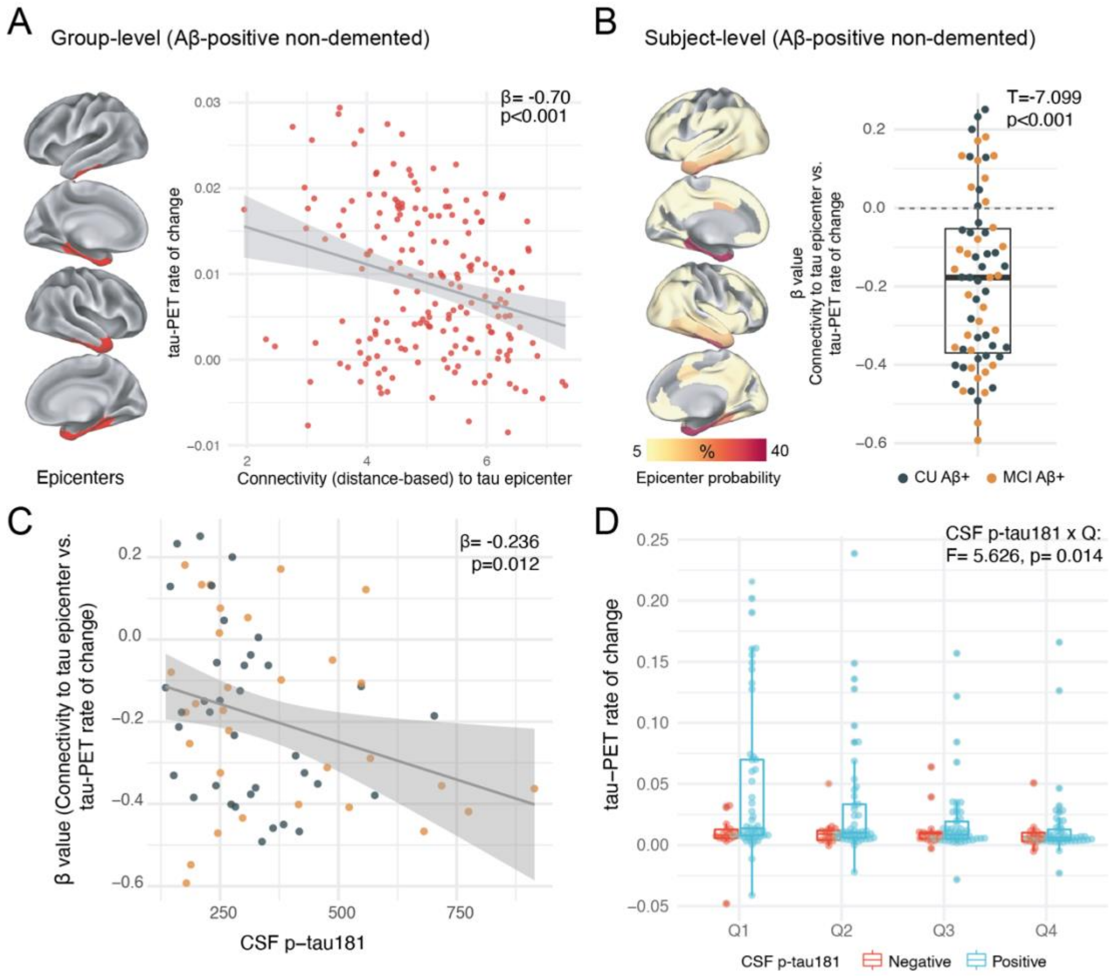

Supplementary Fig. 10. Individualized connectivity-based associations of tau-PET rate of change over time and CSF p-tau181 in Aβ-positive non-demented participants in ADNI
(A) Group-level analysis showing how connectivity to the tau epicenters (projected on the glass brains) relates to tau-PET rate of change across the whole brain. Each dot represents a brain region. Regions more strongly functionally connected to the epicenters have greater rate of tau-PET accumulation **(B)** Repeating the same approach depicted in (A) at the individual level, the values on the glass brains represent the percentage that each region is classified as an epicenter. The box plot shows the individual β-value (n=67) from the correlation between tau-PET rate of change and connectivity-based distance to epicenters across all brain regions **(C)** Scatter plot of the associations between CSF p-tau181 and the β-values of epicenter connectivity to tau-PET rate of change. The expected negative association suggests that higher CSF p-tau181 is associated with the overall pattern of tau-PET change in more functionally connected regions to epicenters **(D)** Average tau-PET rate of change in regions split into quartiles based each region's connectivity to the tau epicenters (Q1 represents top 25% regions with strongest functional connectivity to the epicenters, etc.). In each quartile the dots represent each participant (n=67 in each quartile). All linear regressions performed were two-sided, without adjustment for multiple comparisons and error bands correspond to the 95% confidence interval. In all box plots, the box limits represent the interquartile range and the line depicts the median value.

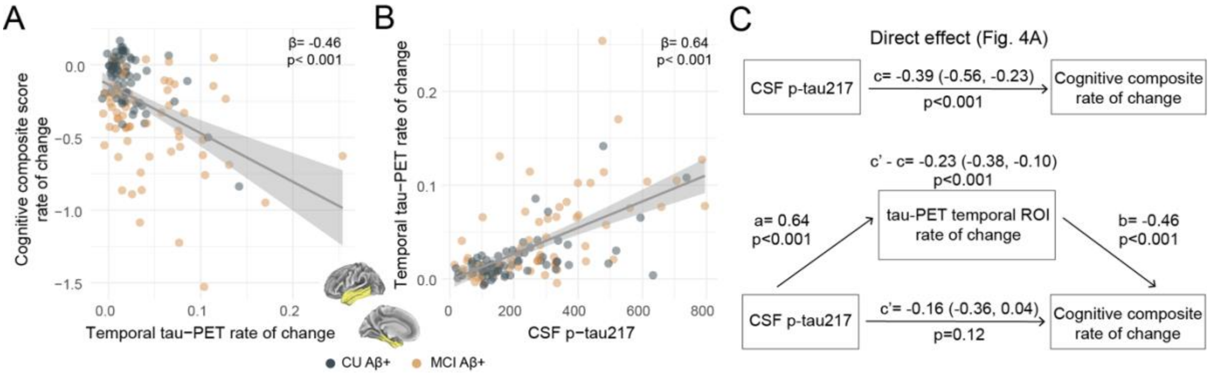

Supplementary Fig. 11. Cognitive decline analyses in Aβ-positive non-demented participants using tau-PET change in the temporal meta-ROI as mediator

Same analyses as in Fig. 4, when using tau-PET rate of change in the temporal meta-ROI (shown in yellow on the brains). Results are the same as when using tau-PET rate of change in Q1 (Fig. 4). (**A**-**B**) Scatter plots of associations relevant to subsequent mediation analyses, beta coefficients from linear regressions adjusting for age, sex and education are reported. (**A**) Association between tau-PET rate of change in the temporal meta-ROI and rate of change on the cognitive composite score. (**B**) Association between CSF p-tau217 and tau-PET rate of change in the temporal meta-ROI. (**C**) Mediation analysis showing a full mediation of tau-PET rate of change in the temporal meta-ROI (58% proportion mediated) on CSF p-tau217 and cognitive decline.

Repeating the same analyses using MMSE rate of change as the cognitive outcome yielded similar results (c'-c= -0.30 [95% CI -0.43, -0.16], p<0.001).

All linear regressions performed were two-sided, without adjustment for multiple comparisons and error bands correspond to the 95% confidence interval.

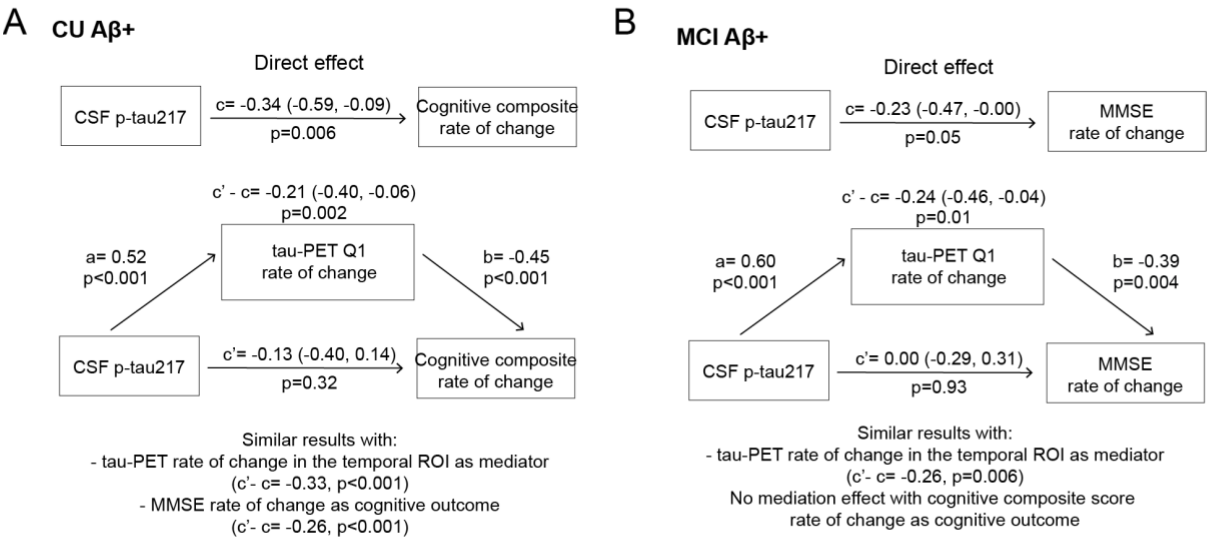

Supplementary Fig. 12. Cognitive decline analyses in Aβ-positive CU and MCI separately

Same mediation analyses as in Fig. 4 in the full sample of non-demented Aβ-positive participants, done here in CU Aβ-positive only **(A)** and MCI Aβ-positive **(B)**. Main analyses were done using tau-PET rate of change in Q1, as shown on the graphs, but also hold when instead using tau-PET rate of change in the temporal meta-ROI. In CU, results were found with rate of change on the cognitive composite score or MMSE. In MCI, results were only found with MMSE rate of change. All linear regressions performed were two-sided, without adjustment for multiple comparisons. Significance of the mediation effect was tested using 1000 bootstrapping iterations.

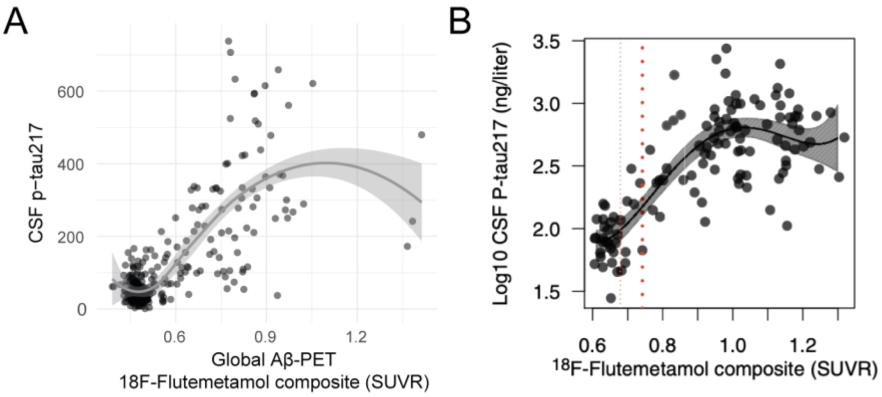

Supplementary Fig. 13. Associations between CSF p-tau217 and Aβ-PET across the AD continuum

(**A**) Associations between global Aβ-PET SUVR (referenced to pons) from flutemetamol and CSF p-tau217. Note that AD dementia patients within BioFINDER-2 undergo lumbar punctures but not Aβ-PET, explaining the gap in highest values. (**B**) Similar association had been shown by our group using a sample of participants that included AD dementia patients, and the same CSF p-tau217 and Aβ-PET measurements. The plateau how CSF p-tau217 evident at high levels of Aβ-PET. Panel adapted from Mattsson-Carlgren et al., *Science Advances*, 2021; **6**: eaaz2387. All error bands correspond to the 95% confidence interval.

Supplementary Table 2. Bivariate correlations in Aβ-positive non-demented participants

	R	p-value
Baseline cognitive composite score vs. CSF p-tau217	-0.23	0.01
Baseline MMSE vs. CSF p-tau217	-0.05	0.56
Baseline cognitive composite score vs. baseline tau-PET temporal ROI	-0.29	<0.001
Baseline MMSE vs. baseline tau-PET temporal ROI	-0.21	0.02
Tau-PET temporal ROI: Baseline vs. rate of change	0.94	<0.001
Baseline tau-PET temporal ROI vs. CSF p-tau217	0.65	<0.001

P-values reported were from two-sided statistical tests, without adjustment for multiple comparisons.
